# Vaccination Conversations on X in Spanish and Catalan: Qualitative Content Analysis

**DOI:** 10.2196/67942

**Published:** 2025-10-14

**Authors:** Agnes Huguet-Feixa, Wasim Ahmed, Eva Artigues-Barberà, Joaquim Sol, Pere Godoy, Marta Ortega Bravo

**Affiliations:** 1 Centre Atenció Primària Rambla Ferran, Atenció Primària, Gerència Territorial de Lleida Institut Català de la Salut Lleida Spain; 2 Fundació Institut Universitari per a la Recerca a l'Atenció Primària de Salut Jordi Gol i Gurina (IDIAPJGol) Barcelona Spain; 3 Grup de Recerca Multidisciplinari en Terapèutica i Intervencions en Atenció Primària (Grup RETICAP) Lleida Spain; 4 Faculty of Medicine University of Lleida Lleida Spain; 5 Hull University Business School Hull United Kingdom; 6 Atenció Primària, Gerència Territorial de Lleida Institut Català de la Salut Lleida Spain; 7 Facultat d’Infermeria i Fisioteràpia Universitat de Lleida Lleida Spain; 8 Unitat de Suport a la Recerca Lleida Fundació Institut Universitari per a la Recerca a l'Atenció Primària de Salut Jordi Gol i Gurina (IDIAPJGol) Barcelona Spain; 9 Institut de Recerca Biomèdica de Lleida, Department of Experimental Medicine University of Lleida Lleida Spain; 10 Gestió de Serveis Sanitaris, Departament de Salut Hospital Universitario Santa Maria Lleida Spain; 11 Centro de Investigación Biomédica en Red de Epidemiologia y Salud Publica Instituto de Salud Carlos III Madrid Spain; 12 Institut de Recerca Biomèdica de Lleida, Applied Epidemiology Research Group University of Lleida Lleida Spain; 13 Centre Atenció Primària Almacelles, Atenció Primària, Gerència Territorial de Lleida Institut Català de la Salut Lleida Spain; 14 Grup de Recerca en Ecografia Clínica d’Atenció Primària (Grup GRECOCAP) Lleida Spain

**Keywords:** antivaccination movement, vaccine refusal, social media, misinformation, vaccination practices, vaccine hesitancy, immunization

## Abstract

**Background:**

The analysis of social networks should be considered by institutions and governments alongside surveys and other conventional methods for assessing public attitudes toward vaccines. X (formerly known as Twitter) has emerged as a significant source for studying vaccine hesitancy.

**Objective:**

The aim of the study is to examine the main arguments and narratives in favor and against vaccination expressed in Spanish- and Catalan-language posts, comments, and opinions on the social media platform X.

**Methods:**

Spanish and Catalan posts were collected from X using NodeXL Pro between March and December 2021, resulting in 479,734 posts. For qualitative analysis, a random subsample of 384 tweets was selected using Cochran’s formula (95% confidence and ±5% margin of error). A bespoke code frame was developed in collaboration with medical and social media experts, and posts were translated into English. Intercoder reliability, assessed on 20% of the sample, yielded 93.4% agreement and a Cohen κ of 0.92.

**Results:**

A total of 479,734 posts were retrieved from 29,706 users. After an inductive review of the data, six themes were identified, which formed the basis of our code frame: (theme 1) vaccine acquisition and distribution, (theme 2) vaccine skepticism and criticism, (theme 3) provaccination stance, (theme 4) global COVID-19 situation, (theme 5) vaccine politics and international relations, and (theme 6) miscellaneous news and posts. Vaccine skepticism and criticism was the most frequent theme (93/384, 24.2%), whereas vaccine politics and international relations was the least (25/384, 6.5%). We observed that while some posts supported vaccination, others expressed concerns about vaccine safety and efficacy, promoted conspiracy theories, disseminated misinformation, or opposed scientific consensus. Challenges related to vaccine acquisition and distribution within specific countries were also identified, along with political and economic factors, such as the politicization of vaccines, which hindered equitable distribution between vaccine-producing and vaccine-needing countries. Additionally, the pandemic’s social impact fostered community support initiatives and solidarity.

**Conclusions:**

Our findings can inform measures to promote vaccine acceptance and reinforce trust in health care systems, professionals, and scientific perspectives, thereby improving vaccination coverage. These insights may serve as a foundation for developing sociopolitical strategies to enhance vaccination management and address future pandemics or new vaccination campaigns.

## Introduction

The internet and social media (SM) have transformed how the public accesses health information [1-4]. X (formerly known as Twitter) has become a significant source for studying vaccine hesitancy, as SM platforms are considered effective tools for communication between individuals and organizations. However, they have also been used to disseminate false information and conspiracy theories about vaccines [5-9].

SM allow individuals to rapidly create and share content on a global scale without editorial oversight. Users can self-select content streams, contributing to ideological isolation. Consequently, antivaccination messages on these platforms are of significant public health concern, as they can lead to vaccine hesitancy [5].

Exposure to antivaccine content on SM has been linked to delays in and refusal of vaccination [10,11]. A decline in vaccination coverage threatens herd immunity and could lead to outbreaks of diseases, such as the measles outbreak in Europe in 2023-2024. Current literature has not yet fully elucidated how the antivaccine movement continues to engage and persuade the public to refuse immunization despite efforts by provaccine advocates to counteract these messages [10].

The spread of antivaccine misinformation is a significant public health challenge influenced by multiple interconnected factors. SM algorithms prioritize engagement-driven content, often amplifying misleading narratives and creating echo chambers that reinforce vaccine skepticism [5,12,13]. On social media, antivaccine advocates address a broader range of topics than provaccine advocates, and previous research has shown that such thematic diversity is associated with higher levels of user engagement. [14-16]. Antivaccine advocates also demonstrate more persuasive and influential narratives than those advocating for vaccines [10]. The rapid dissemination of misinformation, frequently originating from nonexpert sources or commercial entities, further undermines public confidence in vaccines [13,17]. Interaction tends to be higher with antivaccine content than with provaccine content on platforms such as Instagram and Facebook [12,18]. Although antivaccine groups are a minority, they have the potential to be more influential than provaccine groups. Furthermore, undecided groups are not passive; they are the most active participants in the discussion. This dynamic favors interaction between the undecided and antivaccine groups [15].

Conspiracy theories regarding vaccine development and governmental motives exacerbate distrust in public health initiatives [19,20], while ideological polarization limits exposure to scientifically accurate information [13,20]. Previous qualitative studies have reported that provaccine websites focus on accurately conveying evidence-based scientific research on vaccines and government-endorsed vaccination practices, whereas vaccine-skeptic websites aim to build communities of individuals who perceive themselves as affected by vaccines and related practices while questioning the information presented in scientific literature and government documents. Indeed, antivaccine advocates disproportionately emphasize safety concerns while downplaying the preventive benefits of vaccines [4,10].

Recent studies have explored vaccine hesitancy narratives in English-speaking contexts [20-22]; however, less is known about how these discussions unfold in other linguistic and cultural settings, such as Spanish- and Catalan-speaking communities. The objective of this study was to examine the main arguments and narratives in favor and against vaccination expressed in Spanish- and Catalan-language posts, comments, and opinions on the SM platform X. A qualitative approach was essential to enable an in-depth analysis of the discourses, meanings, and sentiments conveyed in these conversations, which cannot be fully captured through quantitative methods alone.

## Methods

### Data Retrieval

Posts were gathered from “X” drawing upon NodeXL Pro. The search string in order to retrieve posts was as follows: “vacunacion OR vacunacio OR vacunas OR vacunes OR antivacunas OR antivacunes OR antivacinacion OR antivacunacio.” These keywords were selected after experimenting with Twitter using the advanced search feature. It was found that, as a collection of keywords, these captured an excellent sample of posts. The way NodeXL Pro captures data means that any of these keywords in their hashtag form, for example, #vacunacion would also have been captured. In addition to this, any replies or mentions to posts with these keywords would also have been captured.

NodeXL Pro previously had access to the Academic Track application programming interface (API) provided by Twitter at the time, which allowed users to retrieve all posts for a topic. However, after the closure of the Academic Track API, affordable academic means to access data became problematic. However, through NodeXL Pro’s new importer, it is possible to capture a random sample of posts across the various months. It is not possible to quantify the sample in terms of the percentage of the entire conversation, and this is further discussed within our limitations.

Our keywords reflect a set of keywords in both Spanish as well as Catalan (spoken in Catalonia, the Balearic Islands, and the Valencian Community). Data were retrieved using NodeXL Pro, which collects a random sample of posts from X based on the specified search terms. However, the underlying parameters of the sampling procedure are not known due to these data not being published and too costly to obtain for most academic research teams. As such, while the dataset reflects activity around the selected queries, it is not possible to determine the precise representativeness of the sample or to know what larger universe of posts it was drawn from. This differs from tools such as the (now discontinued) Twitter Academic API, which provided access to the full stream of eligible posts. These points are further discussed in our limitations.

Duplicate posts were removed from the dataset. However, no bot detection analysis was applied; therefore, automated accounts (bots) may still be present in the sample, and this is further discussed in our limitations.

A total of 479,734 posts were captured (excluding reposts), and the data were retrieved from X between March 2021 and December 2021. We specifically selected 2021 because this corresponds to the second phase of the COVID-19 vaccine rollout where governments began to step up the rollout periods. Table 1 provides an overview of our sample broken down per month.

**Table 1:** Overview of data (tweet volumes and total number of X users).

Month (2021)	Total posts, n	Total X users, n
March	54,837	40,093
April	53,793	39,426
May	53,613	39,114
June	54,499	38,590
July	54,074	40,170
August	54,002	39,303
September	47,141	33,402
October	35,071	25,545
November	35,417	27,944
December	37,287	29,706
Total	479,734	—^a^

^a^Not applicable.

### Qualitative Data Analysis

The data were randomized using a formula in Microsoft Excel, and the team met, reviewed, and discussed the data. A code frame was developed in discussion with medical experts from Spain and expert SM analysts. Although many existing code frames could be used and adapted, due to the highly contextualized Spanish and Catalan languages within the posts, a specific code frame was developed within this context. A sample size was calculated to ensure that a systematic representative sample was analyzed. To determine a sample size sufficient for estimating proportions with a 95% confidence level and a ±5% margin of error, we used Cochran’s formula [23], which yields a sample size of 384 (rounded up). Thus, a random sample of 384 tweets was drawn to provide a representative estimate within ±5% error at 95% confidence. Spanish and Catalan language posts were carefully translated into English to provide example posts for the qualitative analysis. This translation was overseen by a Spanish-speaking expert assisted by translation software such as Google Translate. To assess the consistency of the coding process, a second coder independently coded a sample of 20% (n=77). The intercoder reliability analysis showed a percentage agreement of 93.4%. To account for agreement occurring by chance, Cohen κ was also calculated. The κ coefficient was 0.92, which is considered an indicator of good reliability [24]. Our team further analyzed each of the categories from our code book drawing upon thematic analysis. This enabled the identification of themes, and sub-themes and further interpretative depth

### Ethical Considerations

This study was approved by the ethics and clinical research committee of Fundació Institut Universitari per a la Recerca a l’Atenció Primària de Salut Jordi Gol I Gurina (code 20/221-P). The study was conducted in accordance with the principles of the Declaration of Helsinki. Data were collected using tools that accessed publicly available accounts and domains on the internet. The content obtained through these tools was publicly accessible. Our study drew upon tools such as Grammarly and ChatGPT-4 to assist in improving our language and clarity of expression. However, these tools were not used to generate ideas on their own. Our team maintains full responsbility for the work.

## Results

### Descriptive Overview

**Figure 1:**
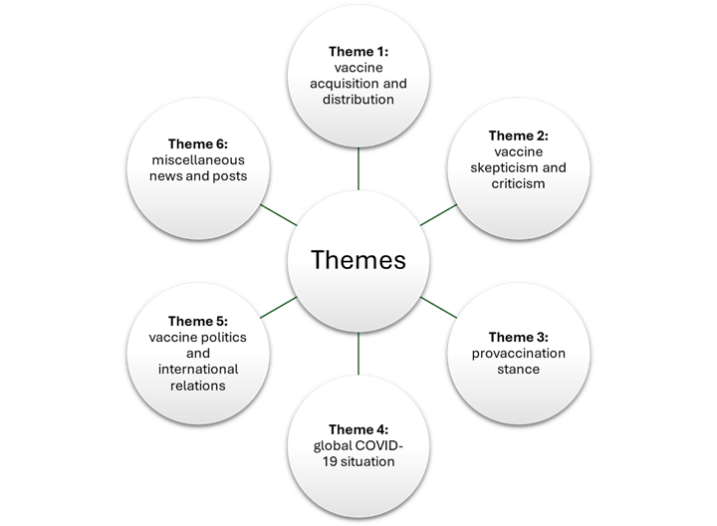
Visual overview of themes to emerge.

Figure 1 provides a visual overview of the 6 key themes that emerged from the data. Table 2 provides an overview of the descriptive statistics and frequencies associated with each of the themes. The table shows the distribution of 384 tweets across 6 themes related to vaccines and COVID-19. The largest share falls under vaccine skepticism and criticism (93/384, 24.2%), while the smallest share is vaccine politics and international relations (25/384, 6.5%).

**Table 2:** Descriptive overview of theme frequency.

Theme	Values, n (%)
Theme 1: vaccine acquisition and distribution	57 (14.8)
Theme 2: vaccine skepticism and criticism	93 (24.2)
Theme 3: provaccination stance	72 (18.7)
Theme 4: global COVID-19 situation	68 (17.7)
Theme 5: vaccine politics and international relations	25 (6.5)
Theme 6: miscellaneous news and posts	68 (17.9)

### Theme 1: Vaccine Acquisition and Distribution

This theme revolved around the logistics and strategies for obtaining and distributing vaccines. Concerns about availability, prioritization, and fairness in vaccine distribution were vital to user discussions.

An example of a post included: “Arrival of the Sinovac vaccines this morning at the CDMX airport. Over 10 million vaccines already in Mexico. Good day!”

The post updated users about the arrival of the Sinovac vaccines in Mexico and appeared celebratory. It highlighted successful vaccine procurement and noted the steady progress in Mexico’s vaccination efforts. The use of “Good day!” communicated a sense of relief or achievement, highlighting the importance of vaccine shipments for public morale and the ongoing battle against COVID-19.

Another illustrative post within this theme included: “Is there something we don’t know about? Aren’t the vaccines arriving in Burgos? Isn’t there staff to administer them? We need an explanation!”

This post reflected concern and uncertainty around the vaccine distribution process in Burgos. The questioning tone, “Is there something we don’t know about?” indicated a perceived lack of transparency or communication from authorities or agencies involved in vaccine management. The post noted the need for clear communication to the public regarding vaccine acquisition, distribution, and administration status.

Other users also questioned the distribution of vaccines in Spain and made claims about missing vaccines. For instance, one user noted:

In the Community of Murcia, more than 600 vaccines disappeared, and there were hundreds of claims of corruption that will not be investigated for now, contrary to those of the City Council. The best way for the PP to continue plundering Spain with impunity is for C’s to disappear.

The author claimed that over 600 vaccines had gone missing in this region, accompanied by numerous allegations of corruption. The statement also carried a strong political accusation, specifically against the People’s Party, accusing them of exploiting the situation for their benefit. The author believed that the People’s Party was engaging in corrupt or self-serving practices at the expense of the public.

Another user made a broader point about charging for vaccines in general:

How many measles or polio vaccines were charged for during the “neoliberal” period? Even giving space to such stupid words is an offense to people’s intelligence. @UserHandle is desperate and has to say whatever it takes to stay afloat.

This post appeared to criticize charging for essential vaccines like measles or polio during a previous political period, which the user labeled as “neoliberal.” The mention of these specific vaccines highlighted a broader conversation about vaccine accessibility and affordability, a point of contention regardless of the pandemic. The user’s frustration indicated criticism of past and present public health and vaccine distribution approaches.

### Theme 2: Vaccine Skepticism and Criticism

Some posts reflected doubts or criticisms about the efficacy, safety, or necessity of vaccines. This theme highlighted the challenges in combating vaccine misinformation and skepticism. Within this theme, 5 subthemes emerged.

One subtheme focused on distrust toward the pharmaceutical industry. Several posts noted concerns over the profit motives of vaccine-producing companies, reinforcing broader skepticism toward the industry. These views were also backed by certain users who were suspicious regarding the rapid development and approval of COVID-19 vaccines.

Another subtheme involved doubts about vaccine efficacy and potential side effects. Some users questioned the level of protection provided, while others voiced concerns about long-term effects, arguing that these risks were either unknown or not fully disclosed.

A third subtheme was the belief in the superiority of natural immunity over vaccine-induced immunity. Many users skeptical of vaccines called for and advocated natural immunity. Moreover, these users expressed skepticism toward external medical interventions, favoring holistic or natural treatments.

Political and ideological perspectives represented another subtheme. Some users perceived vaccination campaigns as mechanisms of governmental control. Discussions branched out, associating vaccination mandates or passports with infringements on personal freedoms.

Finally, misinformation played a crucial role, forming the fifth subtheme. Some posts contained references to conspiracy theories or unfounded claims, such as unverified side effects or misleading arguments in favor of natural immunity. These posts often linked to dubious sources of information. Table 3 provides an overview of post extracts associated with the above subthemes.

**Table 3:** Post extracts for theme 2: vaccine skepticism and criticism.

Subtheme	Post extracts
Distrust in pharmaceutical industry	“Why #vaccine safety and efficacy aren’t known: FDA decided not to require the pharmaceutical companies to track this rigorously once emergency use authorisation had been granted.” “100% agree ... !!! Technically all those, and what, vaccines are nothing more than experimental potions that can poison and lead to death ... !!! 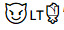 ” “I don’t believe in the ‘virus,’ they have hacked the flu, always ... These are not ‘vaccines,’ they are something else ... I’ll leave it at that ...” “Thanks for the clarification, you are telling me that the population is being vaccinated with experimental vaccines, that they are conducting a clinical trial. And if I remember correctly what was being said was that there were deaths from COVID due to the experimental liquid. 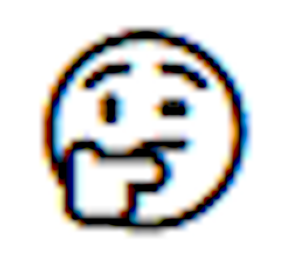 ”
Questioning efficacy and side effects	“These are vaccines that have not been around for long ... We have no idea if they will have any long-term side effects.” “I hope they don’t cause neurological damage, as it has been proven that vaccines can cause autism among other neurological diseases.” “Deaths increased starting from March with the vaccines.” “CDS cleanses the body of heavy metals that come in vaccines. You can contact @UserHandle they use it with immense success.”
Natural immunity advocacy	“My body, my choice. Natural immunity for me. I don’t need the vaccine. Leave our children out of this world-wide experiment.” “Natural immunity is broad, protective, and durable. Unlike vaccine-induced immunity.” “Vaccinated individuals have 27 times higher risk of symptomatic COVID infection compared to those with natural immunity.”
Political and ideological angles	“They plan to provoke as many pandemics as necessary to bring humanity to its knees, until everyone submits to their global control, gives in to genocidal vaccines, to alter DNA, to diminish the world’s population, to sterilise.” “Public order has nothing to do with your uselessness and corruption with the vaccination plan. You won’t achieve anything with blackmail, you bastards.” “Vaccine passports are not ‘our route back to normality.’ They are our route to permanent abnormality, a nightmarish checkpoint ‘Where are your papers?’ society of medical apartheid, discrimination and digital slavery. #StopVaccinePassports #NoNewNormal #DefendFreedom.” “What they call vaccination did not come because of the so-called covid, but covid came to justify what they call vaccination. I just read something similar, and I’m afraid it’s true. Disobedience is the only vaccine. #MiVidaMiOxigeno #DictatureSanitaire #SpainDictatorship.”
Influence of misinformation	“A high-elite athlete who is permanently monitored has never had anything detected and now suddenly has to quit football? An investigation would have to be done. I also suspect that it is yet another case of these vaccines.” “I don’t believe in the ‘virus,’ they have hacked the flu from all our lives ... These are not ‘vaccines,’ they are something else ... I’ll leave it at that ... But I’m not so ‘picky,’ I respect freedom and debate. Thank you and welcome to anyone who fights against this tyranny ... From the heart 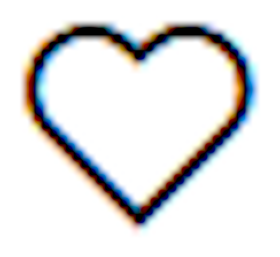 .” “This is worrying. This information has to be known, and then everyone will learn how to act, but when you make things known, you disappear. The body of the doctor who linked vaccines to autism was found floating in the river.” “Shocking video!! The Puppet doctor of the laboratories, who for years was used to testify that vaccines ‘do not produce autism’ ... now he repents and claims the opposite!!!”

### Theme 3: Provaccination Stance

This theme captured posts expressing a positive view toward vaccination, emphasizing its importance in combating the pandemic and urging others to vaccinate. Within this theme, 5 subthemes emerged.

One key aspect was scientific trustworthiness, as users who supported vaccinations emphasized the rigorous testing and validation processes that vaccines underwent. Moreover, users highlighted the endorsements from reputable scientific bodies.

Another subtheme focused on societal responsibility, with an extensive range of users expressing advocacy for vaccination as a communal duty to achieve herd immunity. For these individuals, the interconnectedness of individual actions and broader societal health was essential for preventing the spread of disease.

A third subtheme involved historical successes, as users drew parallels to past vaccination campaigns and their success in eradicating diseases across history. By using historical data and facts, they aimed to validate the effectiveness of vaccines.

Emotional appeals also played a significant role in these discussions. Many users shared personal stories of individuals who were saved by vaccines or those who experienced vaccine-preventable diseases. There was also a range of testimonies shared by frontline workers witnessing the impacts of COVID-19 first-hand.

Finally, debunking myths emerged as a crucial aspect of this theme. In contrast to subtheme 5 from theme 2, which focused on vaccine skepticism and criticism, users in this theme aimed to directly address and dispel common misconceptions about vaccines. Users here sought to use facts and evidence-based research to counter misinformation. Table 4 provides an overview of post extracts associated with the above subthemes.

**Table 4:** Post extracts of theme 3: provaccination stance.

Subtheme	Post extracts
Scientific trustworthiness	“The risk of autism and vaccines is more than disproved, on June 23 a review of 338 studies on the safety of vaccines was published concluding that there is no risk for autism.” “Let’s not forget the damage caused by a false association of vaccination with autism risk. When you have something with an acceptable level of scientific evidence, feel free to share it.” “Guess what did make a difference in controlling the pandemic: vaccines. #FuckIvermectinaParaCOVID #AlwaysTrustScience.” “Vaccines save lives. It’s a simple fact. This isn’t a debate; it’s science. #GetVaccinated #StaySafe.”
Societal responsibility	“The more of us that get vaccinated, the quicker we can return to normal. It’s not just about protecting yourself but also those around you.” “You still know people in your close circle who are not vaccinated. If you were able to bring a non-regular voter, also try to take advantage of the situation and explain to them the need for vaccines. Do not accept the normalisation of such an antisocial action!” “Let’s help in this campaign, for each RT or reply to the cited tweet, UNICEF partners will unlock $1 for UNICEF vaccine programs. Always united #ARMY #VaccinesWork #BTSArmyColombia @UserHandle.”
Historical successes	“For me, the important thing is that vaccines hold up very well for serious diseases, and I’m pretty sure that’s not going to change now.” “The mRNA vaccine story illuminates the way that many scientific discoveries become life-changing innovations: with decades of dead ends, rejections and battles over potential profits, but also curiosity and dogged persistence against scepticism and doubt.” “In the U.S., there have been more than 30 years without people affected by polio thanks to the vaccine.”
Emotional appeals	“The more of us that get vaccinated, the quicker we can return to normal. It’s not just about protecting yourself but also those around you.” “To have the country we want, we have to get vaccinated as we should. Go to your nearest vaccination site and get your first or second dose of a COVID-19 vaccine. #VacúnateYa #JornadaVacúnateYa #VacúnateRD.” “The comptroller @correa_catalino calls on the entire population to get vaccinated against covid-19. The faster you get vaccinated, the faster the country reactivates! #VacúnateRD.” “Received my second dose today! Feeling grateful for the scientists, healthcare workers, and everyone who made this possible. #Vaccinated.”
Debunking myths	“If you’ve not followed the story of how some Americans have turned to a drug called Ivermectin to treat or prevent COVID despite no public evidence it does this, a tweet can’t do it justice. Trust me, it’s worth 7 minutes.” “This delta variant is every infectious disease specialist’s worst nightmare ... There was a myth ... that children were somehow immune ... It has become very clear that children are heavily impacted.” “Catching Covid-19 after being vaccinated isn’t a myth. It happened to me: towards herd immunity: Social distancing + Diagnostic tests + vaccination.”

### Theme 4: Global COVID-19 Situation

Posts under this theme provided a snapshot of the current state of the pandemic worldwide, including discussions of variants, infection rates, and the overall impact of the virus. For example, one user noted: “The pandemic continues to surge in several countries. When will this end? My heart goes out to everyone suffering. 
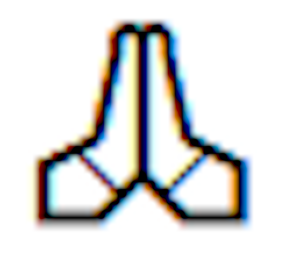
 #COVID19 #StaySafe.”

This post echoed a sentiment of global concern and empathy, highlighting the persistent nature of the pandemic across various countries. The longing to end the crisis and express compassion for those affected spoke to the shared global experience and the challenges many faced.

In another post, a user noted: “Seeing the statistics every day is heartbreaking. Numbers are not just numbers; they represent people. 
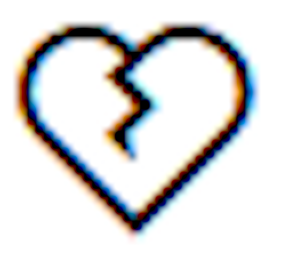
 #COVID19 #PandemicLife.”

This post sought to humanize the numbers, reminding readers of the personal tragedies that comprised the aggregated data and emphasized the crisis’s depth.

Other users reflected on the past; for instance, a user noted: “A year ago, we thought it would be over in a few months. The unpredictability of this virus is something else. #COVID19.”

The post captured the evolving nature of the pandemic and the challenges in predicting its trajectory. The reflection on past expectations versus the current reality underlined the persistent and unpredictable nature of the virus.

### Theme 5: Vaccine Politics and International Relations

Posts within this theme delved into the political dimensions of vaccine distribution, acquisition, and diplomacy. The interactions between nations, the roles of international entities, and geopolitical factors influencing vaccine-related decisions were central to this theme.

For example, one user posted: “The power dynamics in the global health community is evident. Wealthy nations holding the majority of vaccine supplies is a blatant display of neocolonialism. #VaccineEquity.”

This post brought to the forefront the perceived imbalances in global health distribution, particularly around vaccines. Labeling it as “neocolonialism” signified deep-rooted historical connotations where wealth and power were concentrated among a few nations at the expense of others.

Another user posted: “The vaccine diplomacy being played out between superpowers is worrisome. Are we pawns in their global game? #VaccineDiplomacy.”

This post emphasized concerns over the geopolitical strategies major powers undertook, using vaccines as a tool. The phrase “pawns in their global game” suggested that the well-being of citizens might have been secondary to broader political aims.

Another user also noted: “Countries leveraging vaccines for political favors sets a dangerous precedent. Public health should transcend politics. #VaccinePolitics.”

The post highlighted the potential risks when vaccines became intertwined with political agendas. The call for public health to “transcend politics” was a plea for prioritizing humanity and well-being over political maneuvers.

In the broader context of vaccine policies, a user also noted: “I was in a public school 40 years ago, and even then, a vaccination card was required for enrolment, and it was perfectly normal.”

This post reflected on past public health practices in schools, where the user shared their experiences with vaccination cards as a regular part of enrollment procedures. It suggested a historical acceptance and understanding of the importance of vaccinations for public health. The post indirectly supported the idea that vaccinations have been an integral and uncontroversial part of societal norms, promoting the general well-being and safety of communities long before the advent of COVID-19.

### Theme 6: Miscellaneous News and Posts

This theme covered general updates, news, and other miscellaneous information about vaccines and the pandemic. For instance, a user noted: “It’s heartwarming to see communities come together, setting up local vaccine drives and educating each other. Unity in times of crisis. #CommunityResponse.”

This post highlighted the positive aspects of collective behavior, showcasing the power of community resilience and cooperation during trying times. The emphasis on localized initiatives and educational programs highlighted the presence of grassroots movements, which were indicative of community-driven efforts to foster systemic change from the bottom up, rather than relying on top-down institutional mandates.

Another user also noted: “In a world where everyone has an opinion, it’s tough to sift through the noise. Wish there was more emphasis on science in public discourse. #DrowningInOpinions.”

This post examined the challenges of information overload in the digital age. The desire for a stronger emphasis on science indicated a concern about misinformation or the dilution of factual discourse in the public realm.

Another user also shared the following post: “Just read a story about a small town turning their community center into a space for COVID survivors to share stories and heal. It’s not all about stats and data; it’s about people and their journeys. #HumanSideOfPandemic.”

This post brought attention to the human aspects of the pandemic beyond just numbers and data. It highlighted the importance of emotional healing and community support in navigating the crisis. The resilience and adaptability of communities were evident in their self-organized responses to crises. Local initiatives, whether vaccine drives or support groups, played a crucial role in bolstering societal well-being.

Another example of a broader post included the following: “Vaccines are arriving, restrictions are in place, so the system doesn’t collapse. They sold you the idea that the goal is to destroy the national government at all costs, even if it means risking your own life. It seems to me that you are being used.”

This post discussed the broader dynamics around vaccination and public health measures and highlighted the wide type of conversations within this theme. The user seemed to criticize a narrative that accused the government of implementing vaccination and restriction policies for ulterior motives, suggesting instead that these measures were meant to protect public health and prevent the health care system overload. While they involved vaccines, these discussions often revealed deeper societal and political tensions and were illustrative of the complex environment in which health policy was debated and implemented.

## Discussion

We conducted a qualitative content analysis of comments and opinions from posts in Catalan and Spanish regarding vaccination and antivaccination in X, identifying 6 predominant themes:

### Theme 1: Vaccine Acquisition and Distribution

The spectrum of experiences highlighted in the posts illustrated the challenges different nations and regions face in their vaccination campaigns. While some celebrated milestones, others encountered uncertainties and sought answers, emphasizing the uneven landscape of global vaccine distribution. Transparent and effective communication emerges as a fundamental element in vaccination campaigns. Ambiguity may foster confusion, mistrust, or even the spread of misinformation [25]. Ensuring that the public remains informed and confident requires clear communication channels between governing bodies and the population [26]. The Strategic Advisory Group of Experts (SAGE) Working Group on Vaccine Hesitancy categorized vaccine hesitancy using a matrix of contextual, individual or social, and vaccine- and vaccination-specific issues [27]. This theme of vaccine acquisition and distribution is classified as a contextual determinant that influences vaccine acceptance or rejection, as defined by the SAGE Working Group [28,29]. Additionally, according to the World Health Organization’s (WHO’s) “3Cs” (confidence, complacency, and convenience) model, convenience in vaccines and in the health care system constitutes 1 of the 3 main determinants of vaccine hesitancy [29]. It has been demonstrated that proper vaccine distribution prevents significant morbidity and mortality [30], as evidenced by the fact that the implementation of the WHO’s Expanded Programme on Immunization has averted 154 million deaths over the past 50 years [31].

### Theme 2: Vaccine Skepticism and Criticism

Some posts reflected doubts or criticisms regarding vaccines’ efficacy, safety, or necessity. These posts focused on skepticism toward the COVID-19 vaccine, which was more prevalent at the time, as well as skepticism about vaccines outside the context of COVID-19. In these posts, the efficacy of vaccines was questioned, and secondary effects were attributed to the vaccines or their components, alongside conspiracy theories. The skepticism highlighted in these posts extended beyond the vaccines themselves, challenging the entire COVID-19 narrative, from the existence of the virus to global response strategies. Such deep-rooted skepticism presents a significant barrier to the universal acceptance of health guidelines and interventions. The emotive nature of these views suggests that simply countering them with facts may not be sufficient. Addressing these perspectives requires a comprehensive strategy that disseminates accurate information and rebuilds trust. The challenge lies in effectively communicating health information to a divided audience. These findings are consistent with previous studies showing that vaccine-skeptic websites emphasize safety concerns and question the information presented in scientific literature and government documents [4,10]. Confidence in vaccines is 1 of the 3 main determinants of vaccine hesitancy described by the WHO’s “3Cs” model [29]. Vaccine hesitancy is complex and context-specific, varying across time, place, and vaccines. Vaccine hesitancy may be influenced by a combination of sociodemographic and socioeconomic factors [32-34], psychological perceptions [33,35,36], concerns about vaccine safety and efficacy [33,35,37,38], trust and confidence [32,35,37,39], information and communication [34,35,40], as well as cultural, social, and political influences [22,34,40]. A prepandemic publication analyzing the psychological factors that might motivate people in 25 countries to refuse vaccination showed that the best predictors of refusal were the following: high levels of conspiracy beliefs, low tolerance of perceived infringement of personal freedom, aversion to needles or blood, and religious beliefs [41]. A review during pandemics highlighted the complexity of factors related to acceptance or refusal of vaccines including demographic factors (ethnicity, age, sex, pregnancy, education, and employment), accessibility and cost of vaccines, personal responsibility and risk perceptions, trust in health care authorities and vaccines, safety and efficacy of new vaccines, and lack of information or misinformation [34]. These factors do not act in isolation but are interconnected with a range of contextual, individual, and vaccine-specific influences, shaping vaccination decisions, as highlighted in the WHO 3Cs model [27,29].

Some posts in our study linked vaccines or their components to neurological damage and autism, referencing the 1998 study by Wakefield et al [42], which falsely claimed an association between the measles-mumps-rubella vaccine and autism. The *Lancet* later published a retraction of the paper due to the false claims it contained [43]. Despite this, the paper of Wakefield et al [42] continues to accumulate a significant number of citations [44] and was linked to a sharp decline in childhood measles-mumps-rubella vaccination rates, leading to subsequent measles outbreaks [45].

### Theme 3: Provaccination Stance

These posts captured the essence of a movement that was not only rooted in scientific belief but also emphasized community welfare and solidarity. The emphasis was not solely on personal health but on the collective well-being of society. The concept of collective responsibility is 1 of 5 factors that affect people’s perception of vaccines, along with confidence (trust in vaccine efficacy and safety), complacency (perception of the risk of the disease), calculation (weighing the risks and benefits of vaccines), and constraint (accessibility of information about the vaccine) [46]. These are part of the 5Cs model [46], which extends from the 3Cs model introduced by the WHO SAGE Working Group [29].

Although the provaccination messages in our study were not predominant, they reflected trust in the scientific community and its advancements, particularly during the pandemic. This indicated a broader sentiment that valued empirical evidence and the progress of modern medicine. In an era where misinformation can spread rapidly, these posts acted as counternarratives that upheld scientific principles. However, the influence of provaccine content is often limited by its emotional or narrative appeal [16,47], the network dynamics of antivaccine messages [12,15,48], the spread of misinformation [49], and the challenges of effectively communicating scientific consensus [50,51]. Provaccine messages that rely solely on scientific knowledge tend to be less engaging [16,47], whereas antivaccine messages often use emotional narratives such as government conspiracies or alleged vaccine harm, which resonate more strongly and generate higher engagement [4,10,52]. SM platforms often create echo chambers, where users are primarily exposed to information that aligns with their existing beliefs [12]. Antivaccine clusters are more interconnected and engage more frequently, reinforcing their views, while provaccine clusters are more fragmented and peripheral [48]. A previous study published in 2020, which analyzed global Facebook users, indicated that although antivaccine groups were a minority, they may have been more influential than provaccine groups [15]. Antivaccine content often simplifies complex issues, making them more accessible and easier to understand, which can lead to its wider dissemination and acceptance [49]. Scientific consensus can reduce concerns about vaccines, but it is not always effective in changing the attitudes or intentions of those who distrust scientists [50,51]. Provaccine messages must convey confidence and reassurance, both informationally and emotionally, to effectively counter antivaccine narratives [10,52].

### Theme 4: Global COVID-19 Situation

The global reach of the pandemic meant that people across different countries and cultures were sharing remarkably similar experiences and challenges. This collective ordeal has the potential to foster international solidarity, even as it also reveals disparities in resources and responses. The daily barrage of statistics and news not only informed but also profoundly affected many. The emotional impact of the continuous stream of information was especially evident. The posts highlighted the shared challenges, the emotional toll, and the evolving nature of the pandemic, emphasizing the need for global collaboration, mental well-being, and adaptability. The WHO published a scientific brief in 2022, highlighting the impact of the COVID-19 pandemic on the prevalence of mental health symptoms and mental disorders as well as the effectiveness of psychological interventions in preventing or reducing mental health problems and/or maintaining access to mental health services [53].

### Theme 5: Vaccine Politics and International Relations

The posts in our study showed that during the COVID-19 vaccination process, it became evident that political and public health factors were interconnected with the economy. The disparities in vaccine distribution and access among nations were not just logistical but deeply political [54]. The advent of “vaccine diplomacy” demonstrated how crucial medical supplies could be leveraged for geopolitical influence [55,56]. Such practices could potentially jeopardize genuine public health efforts by introducing additional layers of strategic considerations. The politicization of vaccines could set precedents for future global health crises. The unequal distribution of vaccines during the pandemic was also evident in other published papers, which highlighted how manufacturing countries and those reliant on imports vied for diplomatic advantage. The former sought to achieve hegemony, while the latter aimed to secure vaccine supplies [55,57-60].

### Theme 6: Miscellaneous News and Posts

Given the wide-reaching impact of the COVID-19 pandemic, it was unsurprising that it generated a broad range of discussions. This theme also captured the miscellaneous conversations, which, while not directly related to vaccines or governmental responses, shed light on the narratives that shaped public sentiment. Moreover, the wide range of personal stories and experiences that arose during such a global crisis underlined the importance of listening to and understanding these voices as we reflected on the pandemic. It served as a reminder that each post represented a person with their unique journey and their challenges. The resilience and adaptability of communities were evident in their self-organized responses to crises. Other publications also showed that community support initiatives were implemented during the pandemic to enhance connectivity, reduce isolation, and facilitate the sharing of critical information and resources [61,62]. Based on our study’s findings, we believe that strengthening trust in the health care system and combating misinformation through community actions are key to reducing vaccine hesitancy.

### Limitations and Future Research

A limitation of our study is that the qualitative content analysis of Spanish posts focused specifically on the X network in 2021, during the COVID-19 pandemic, and our results reflect opinions influenced by the pandemic. Nevertheless, our study reveals reasons for vaccine acceptance or hesitancy that had been previously described by the WHO before the pandemic, such as trust in the health care system and scientific attitudes, as well as the efficacy and safety of vaccines. These factors continue to be relevant in the current context. Another limitation of the study was the inherent recruitment bias associated with restricting participation to individuals with internet access. Several studies have analyzed public opinions or sentiments about vaccines on X using artificial intelligence. These publications suggest that institutions and governments should consider analyzing social networks with artificial intelligence alongside surveys and other conventional methods for assessing public attitudes toward vaccines [6,10,63-65]. Future research could aim to extend the time periods and examine discussions on X in relation to other SM platforms. Future research could also seek to combine qualitative and quantitative approaches in order to gain a statistical understanding of the topic. This could include user demographics as well as engagement metrics. Moreover, it is also important to note limitations ascertaining to the analysis of a single platform. This can introduce platform dynamics, such as echo chambers, and algorithmic bias, which will consequently impact the results and themes that emerge from a platform. There are also limitations in regards to the absence of non-Spanish or Catalan speakers as well as offline populations. In regard to language, due to limitations in geocoded data from Twitter, our data captured posts sent in Spanish or Catalan from around the world. Future research could seek to isolate and compare different regions. A further limitation of this study is that we did not implement bot detection methods. As a result, some automated accounts (bots) may remain in the dataset, which could have influenced the nature and frequency of certain themes. Future work could incorporate bot-detection approaches to strengthen the robustness of analyses. A further important limitation concerns the use of NodeXL Pro for data retrieval. NodeXL Pro provides a random sample of posts, but the sampling parameters are not available due to the cost and complexity of obtaining data. This means we cannot determine the exact population of posts from which our dataset was drawn. While this does not affect the qualitative thematic insights, it does limit the generalizability of descriptive statistics. Future research could build on our inductive findings by mapping them onto established frameworks such as the WHO’s 3Cs model. This would allow the exploration of how context-specific themes identified in Spanish and Catalan discourse align with or diverge from broader patterns of vaccine hesitancy. Despite these limitations, our study provides valuable insights into Spanish and Catalan vaccine opinions, though caution is warranted when extrapolating the findings to the entire population.

### Conclusions

Our study enabled the analysis of Spanish- and Catalan-language posts concerning opinions for and against vaccination, the social and political impact of the COVID-19 pandemic, and the acquisition and distribution of vaccines. We observed that while some posts supported vaccination, others raised concerns about vaccine safety and efficacy, promoted conspiracy theories, spread misinformation, or opposed scientific consensus. Challenges related to vaccine acquisition and distribution within certain countries were also identified, while political and economic factors, such as the politicization of vaccines, hindered equitable distribution between vaccine-producing countries and those in need. Additionally, the social impact of the pandemic was noted to foster community support initiatives and solidarity. Our findings will be useful for implementing measures to facilitate vaccine acceptance and strengthen trust in the health care system, health care professionals, and scientific perspectives, thus improving current vaccination coverage. Public health authorities should study and counteract antivaccine messages through social networks. Additionally, these insights could serve as a starting point for developing sociopolitical strategies to enhance vaccination management and address potential future pandemics or new vaccination campaigns.

## Abbreviations

3C: confidence, complacency, and convenience
API: application programming interface
SAGE: Strategic Advisory Group of Experts
SM: social media
WHO: World Health Organization

## Data Availability

The datasets generated or analyzed during this study are available from the corresponding author on reasonable request.
